# Correction to: Phosphorylation of pericyte FAK‑Y861 affects tumour cell apoptosis and tumour blood vessel regression

**DOI:** 10.1007/s10456-021-09802-9

**Published:** 2021-07-04

**Authors:** Delphine M. Lees, Louise E. Reynolds, Ana Rita Pedrosa, Marina Roy-Luzarraga, Kairbaan M. Hodivala-Dilke

**Affiliations:** Adhesion and Angiogenesis Laboratory, Centre for Tumour Microenvironment, Barts Cancer Institute – a CR-UK Centre of Excellence, Queen Mary University of London, John Vane Science Centre, Charterhouse Square, London, EC1M 6BQ UK

## Correction to: Angiogenesis 10.1007/s10456-021-09776-8

In the original publication, Fig. 1 and Fig. 4 were published incorrectly. The correct figures are provided in this correction.

**Fig. 1:**
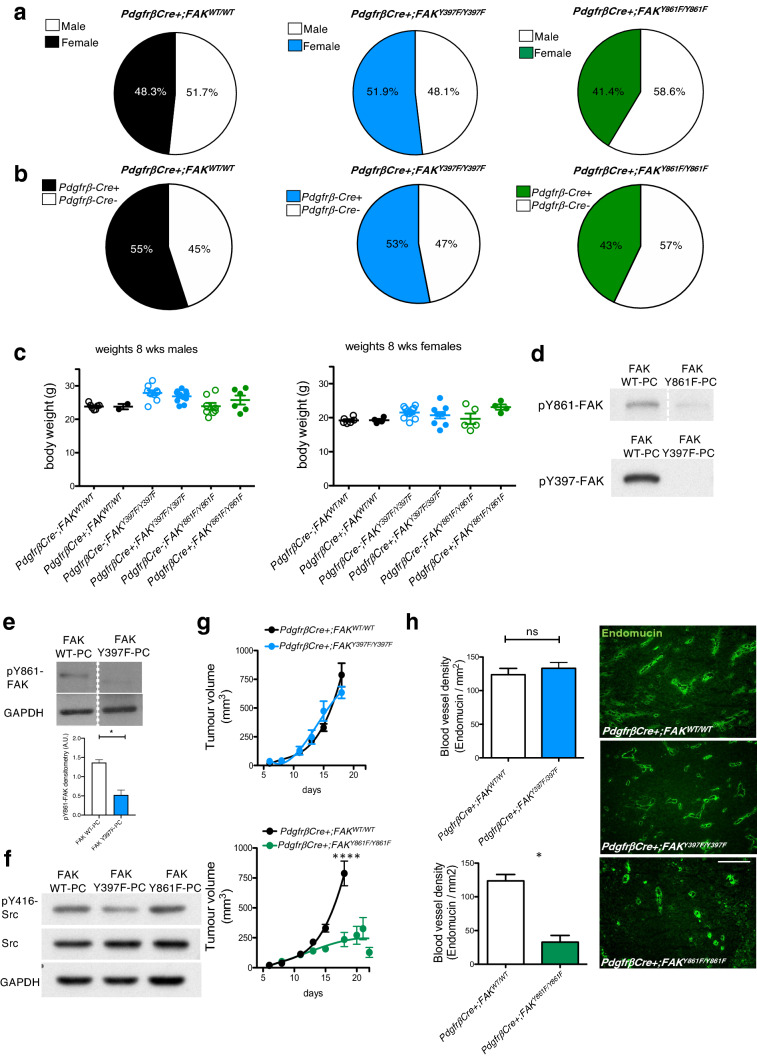
LLC tumour growth and angiogenesis are reduced in PdgfrβCre + ;FAK^Y861F/Y861F^ mice. **a** *PdgfrβCre* + *;FAK*^*WT/WT*^, *PdgfrβCre* + *;FAK*^*Y397F/Y397F*^ and *PdgfrβCre* + *;FAK*^*Y861F/Y861F*^ mice were born at normal male;female ratios; **b** Mendelian ratios with **c** similar body weights. Pie chart in **a** represents percentage male:female ratio, in **b** represents % Cre + and Cre- mice born to each genotype (*n* = 60 mice/genotype). **d** Western blotting of primary pericytes isolated from *PdgfrβCre* + *;FAK*^*WT/WT*^, *PdgfrβCre* + *;FAK*^*Y397F/Y397F*^ and *PdgfrβCre* + *;FAK*^*Y861F/Y861F*^ mice confirmed reduced levels of p-Y397 and pY861-FAK in FAKY397F and FAKY861F pericytes, respectively. **e** pY861-FAK levels are significantly reduced in Y397F pericytes, *n* = 2 independent lysates/genotype. Bar chart represents mean pY861-FAK levels + sd. *P = 0.0155. GAPDH act as loading control. **f** pY416-Src levels are significantly reduced in Y397F-FAK pericytes. Blots shows pY416-Src, total Src and GAPDH loading control. **g** In vivo tumour growth was significantly reduced only in *PdgfrβCre* + *;FAK*^*Y861F/Y861F*^ mice. Graphs represent mean tumour volume ± s.e.m.; *n* = 15 *PdgfrβCre* + *;FAK*^*WT/WT*^ mice, 14 *PdgfrβCre* + *;FAK*^*Y397F/Y397F*^ mice and 11 *PdgfrβCre* + *;FAK*^*Y861F/Y861F*^ mice. ****P < 0.0001. Two-sided Mann–Whitney *U* rank sum test. **h** Tumour blood vessel density was significantly reduced only in *PdgfrβCre* + *;FAK*^*Y861F/Y861F*^ mice. Bar charts represent mean blood vessel density + s.e.m. *P = 0.0498; ns, not significant; *n* = 6 *PdgfrβCre* + *;FAK*^*WT/WT*^ tumours, 6 *PdgfrβCre* + *;FAK*^*Y397F/Y397F*^ tumours and 5 *PdgfrβCre* + *;FAK*^*Y861F/Y861F*^ tumours. Two-sided Student’s *t*-test. Representative endomucin stained LLC tumour sections are shown for each genotype. Scale bar, 50 μm

**Fig. 4:**
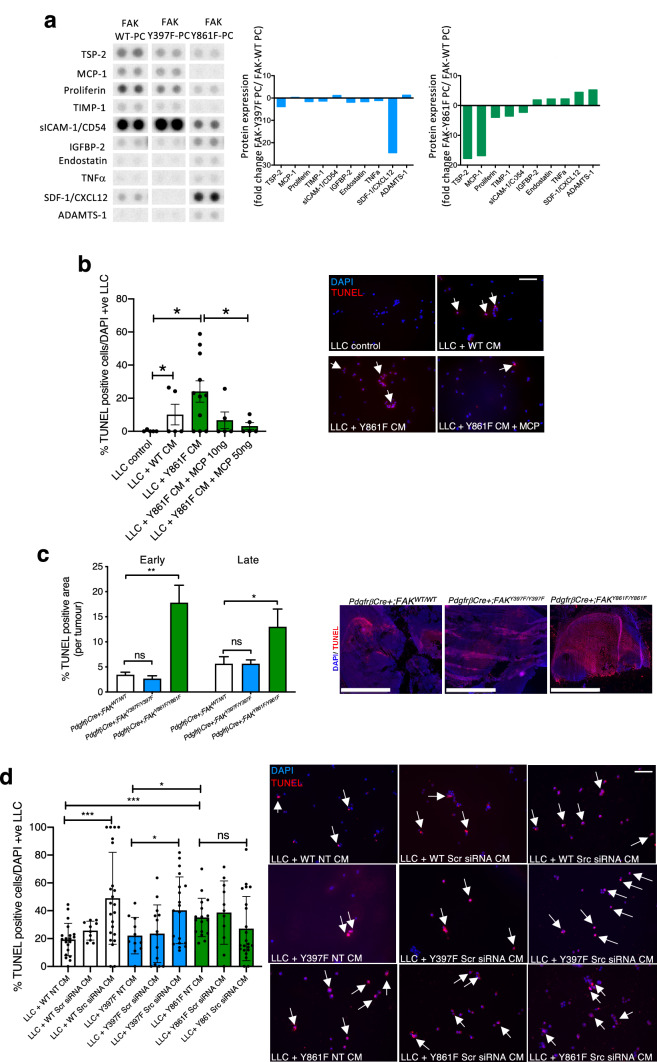
FAK-Y861F pericytes induce apoptosis in LLC tumour cells. **a** R&D proteome profiler array using lysates from FAK-WT, FAK-Y397F and FAK-Y861F pericytes. Representative dots of differentially expressed proteins are given. Bar charts show mean fold change in protein expression relative to levels in WT. *N* = 2 dots from 1 experiment. **b** Lewis lung carcinoma (LLC) cells incubated with conditioned medium (CM) from pericytes plus or minus recombinant MCP-1. Treatment with FAK-Y861F pericyte CM increased LLC apoptosis, compared with CM from FAK-WT pericytes. This effect was rescued upon treatment with MCP-1 (10 and 50 ng/ml). Bar chart represents % TUNEL-positive cells + s.e.m. Representative images show effect of CM and MCP-1 on LLC cells. *Arrows*, TUNEL-positive cells. *P < 0.05. One-way ANOVA. *N* = 5–11 fields of view/genotype. Scale bar, 500 μm. **c** Early and late stage tumours from *PdgfrβCre* + *;FAK*^*Y861F/Y861F*^ mice had significantly larger TUNEL-positive areas than tumours from *PdgfrβCre* + *;FAK*^*WT/WT*^ and *PdgfrβCre* + *;FAK*^*Y397F/Y397F*^ mice. Bar chart shows % TUNEL-positive area/tumour + s.e.m. **P = 0.0012, *P = 0.0464; ns, not significant; *n* = 6 *PdgfrβCre* + *;FAK*^*WT/WT*^ mice, 8 *PdgfrβCre* + *;FAK*^*Y397F/Y397F*^ mice and 6 *PdgfrβCre* + *;FAK*^*Y861F/Y861F*^ mice. Two-way ANOVA. Representative images show TUNEL-positive staining in tumours. Scale bar, 2.5 mm. **d** LLC cells incubated with CM from pericytes treated with Src siRNA, non-specific scrambled siRNA (Scr) or transfection reagent (NT). CM from non-treated (NT) Y861F had a significantly higher effect on LLC apoptosis compared with CM from either WT or Y397F pericytes. Knockdown of Src in both WT and Y397F pericytes significantly increased LLC apoptosis. Src knockdown in Y861F pericytes did not increase LLC apoptosis above control levels. Bar chart represents % TUNEL-positive cells ± s.e.m. ***P = 0.0006, *P = 0.0196 One -way ANOVA. ***P = 0.0006 WT NT vs. 861F NT, *P = 0.03 (Y397F NT vs. Y861F NT). Two-sided students *t* test. *N* = 10–23 fields of view. Scale bar, 500 μm; *arrows* in **b** and **d** indicate TUNEL-positive cells

The original article has been corrected.

