# Supplementary figures and images for: Phosphorylation of pericyte FAK-Y861 affects tumour cell apoptosis and tumour blood vessel regression
Source: Angiogenesis. 2021 Mar 17;24(3):471–82. doi: 10.1007/s10456-021-09776-8 (PMC8292267; doi:10.1007/s10456-021-09776-8)

**a**

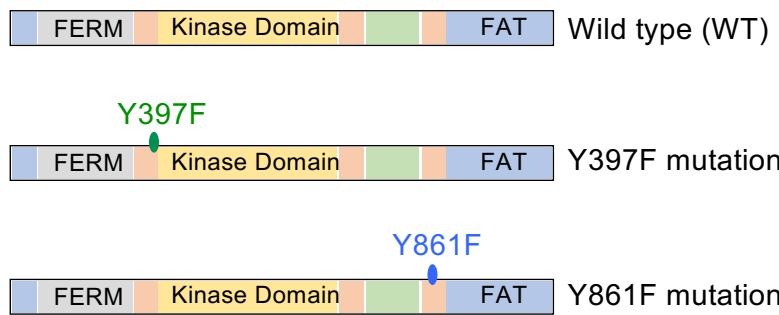

**b**

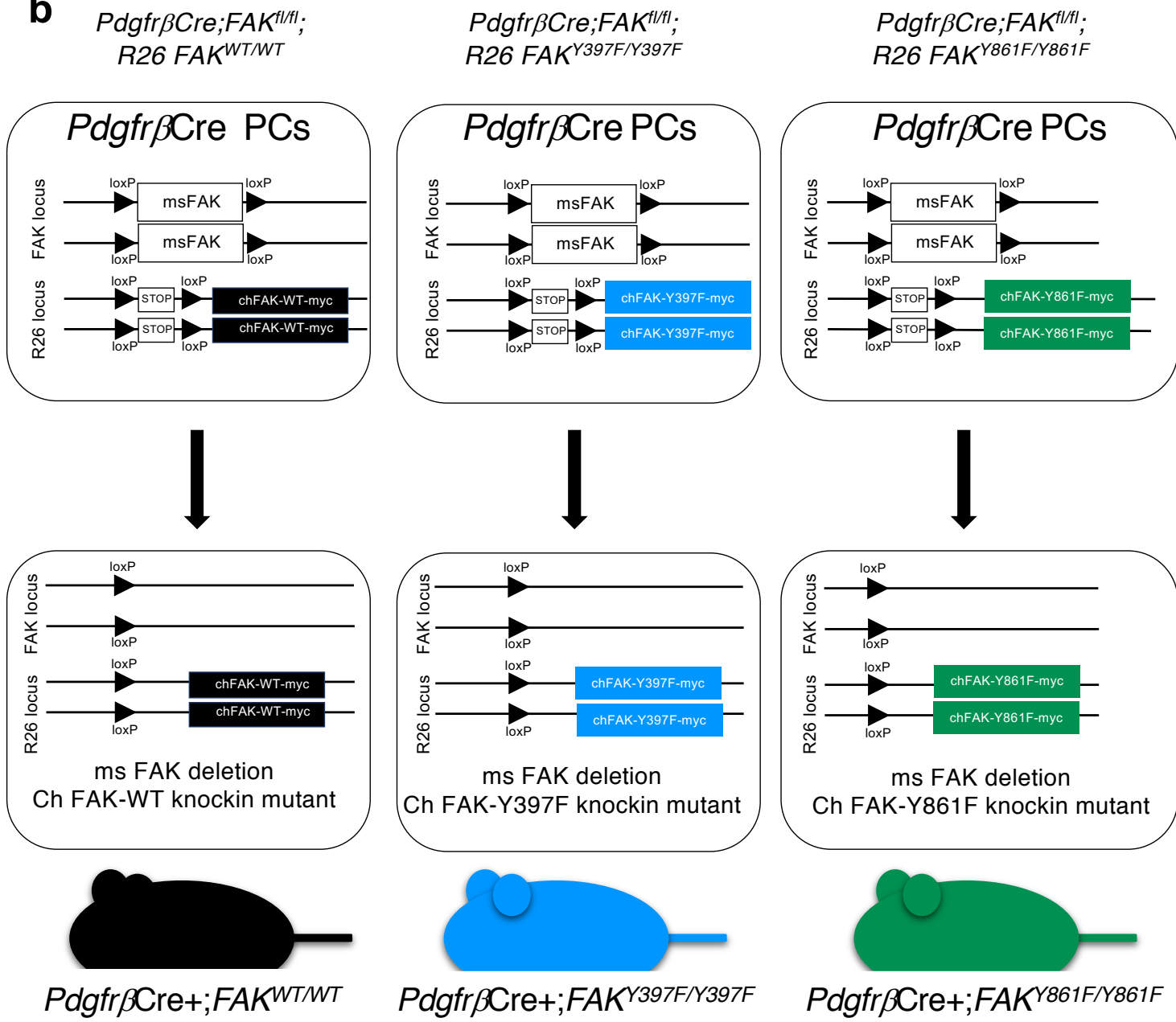

**c**

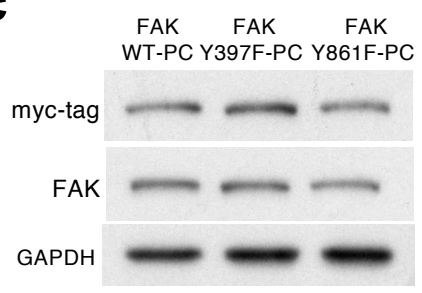

Supplement: Supplementary file 1 — Supplementary file1 (PDF 391 KB) [file 10456_2021_9776_MOESM1_ESM.pdf]

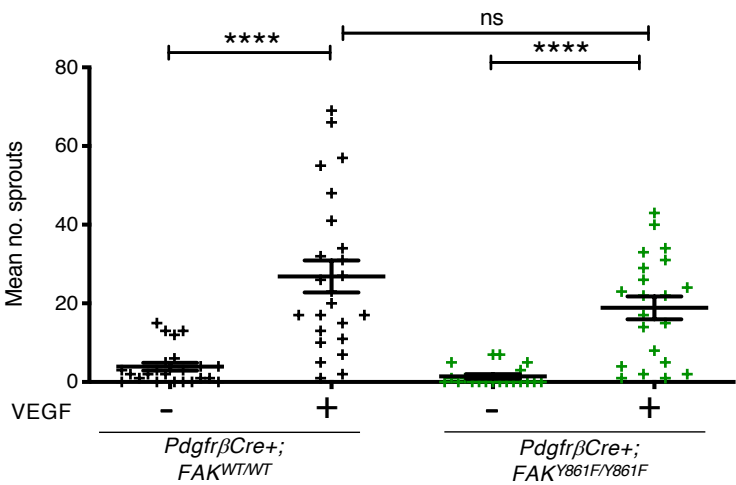

Supplement: Supplementary file 2 — Supplementary file2 (PDF 55 KB) [file 10456_2021_9776_MOESM2_ESM.pdf]

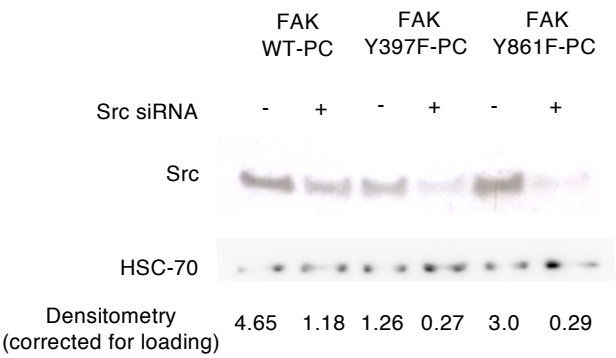

Supplement: Supplementary file 3 — Supplementary file3 (PDF 568 KB) [file 10456_2021_9776_MOESM3_ESM.pdf]
